# The effects of arbuscular mycorrhizal fungi on glomalin-related soil protein distribution, aggregate stability and their relationships with soil properties at different soil depths in lead-zinc contaminated area

**DOI:** 10.1371/journal.pone.0182264

**Published:** 2017-08-03

**Authors:** Yurong Yang, Chuangjun He, Li Huang, Yihui Ban, Ming Tang

**Affiliations:** 1 School of Environment, Key Laboratory of Ecological Restoration & Ecosystem Management, Northeast Normal University, Changchun, China; 2 Institute of Grassland Science, Northeast Normal University, Key Laboratory of Vegetation Ecology, Ministry of Education, Changchun, China; 3 College of Forestry, Northwest A&F University, Yangling, Shaanxi, China; 4 College of Forestry and Landscape Architecture, South China Agricultural University, Guangzhou, China; Sun Yat-Sen University, CHINA

## Abstract

Glomalin-related soil protein (GRSP), a widespread glycoprotein produced by arbuscular mycorrhizal fungi (AMF), is crucial for ecosystem functioning and ecological restoration. In the present study, an investigation was conducted to comprehensively analyze the effects of heavy metal (HM) contamination on AMF status, soil properties, aggregate distribution and stability, and their correlations at different soil depths (0–10, 10–20, 20–30, 30–40 cm). Our results showed that the mycorrhizal colonization (MC), hyphal length density (HLD), GRSP, soil organic matter (SOM) and soil organic carbon (SOC) were significantly inhibited by Pb compared to Zn at 0–20 cm soil depth, indicating that HM had significant inhibitory effects on AMF growth and soil properties, and that Pb exhibited greater toxicity than Zn at shallow layer of soil. Both the proportion of soil large macroaggregates (>2000 μm) and mean weight diameter (MWD) were positively correlated with GRSP, SOM and SOC at 0–20 cm soil depth (*P* < 0.05), proving the important contributions of GRSP, SOM and SOC for binding soil particles together into large macroaggregates and improving aggregate stability. Furthermore, MC and HLD had significantly positive correlation with GRSP, SOM and SOC, suggesting that AMF played an essential role in GRSP, SOM and SOC accumulation and subsequently influencing aggregate formation and particle-size distribution in HM polluted soils. Our study highlighted that the introduction of indigenous plant associated with AMF might be a successful biotechnological tool to assist the recovery of HM polluted soils, and that proper management practices should be developed to guarantee maximum benefits from plant-AMF symbiosis during ecological restoration.

## Introduction

With the rapid development of the industrialization and urbanization process over the past several decades, soils contaminated with heavy metals (HMs) have become major issues in many developing countries [1, 2]. Elevated soil HMs can be attributed to a number of human activities such as mining, smelting, electroplating, fuel production, gas exhausts and intensive agriculture [3]. Due to less advanced technologies and more emphasis on economic growth, mining and smelting operations for metallic ores have been the dominant sources of soil HMs and subsequently caused severe environmental pollution in China [4]. Unlike carbon-based organic pollutants, HMs cannot be degraded or destroyed easily and therefore tend to accumulate in various ecosystems over a long period of time [5]. Considerable amounts of HMs not only have long-term hazardous impacts on soil function and quality, microbial activity and diversity, plant growth and metabolism, but also threaten the health and life of animals and human beings through the food chain [1, 6]. However, some plants develop many detoxification and tolerance mechanisms that are able to survive, grow and reproduce without obvious toxicity symptoms in HM polluted soils [7]. Phytoremediation refers to the use of these plants to remove HM pollutants from soils in a non-polluting and cost-effective way [8]. Whereas, most of these plants are not good for phytoremediation due to their slow growth rate and small biomass, thus will take very long time for effective remediation of a site [4].

Arbuscular mycorrhizal fungi (AMF) are ubiquitous soil organisms that form mutual symbiotic association with over 80% terrestrial plants, providing a direct physical link between soil and plant roots [9]. AMF have been shown to promote plant growth and HM tolerance by increasing plant access to relatively immobile nutrients [10], enhancing plant growth hormones [11], influencing the uptake and distribution of HMs in plant tissues [12, 13], and improving the conditions of rhizosphere soil micro-environment [14]. Therefore, AMF can serve as a potential biotechnological tool to increase the phytoremediation efficiency of HM contaminated soils [15, 16]. The influence of AMF on improving soil quality and structure in HM polluted area has been largely neglected, although numerous studies have revealed beneficial role of AMF in phytoremediation from symbiont perspective [17, 18].

AMF can contribute to soil aggregate stability directly by a physical effect of a network around soil particles, and indirectly by the hyphal exudation of an iron-containing, heat stable glycoprotein (extracted at 121°C) named glomalin as an aggregate binding agent [19, 20]. Glomalin has been operationally defined as glomalin-related soil protein (GRSP) by extraction and detection conditions from soil, and it is detected in large amounts in diverse ecosystems [20]. The sticky GRSP acts as biological glue, helping to bind soil tiny particles into small aggregates of different sizes [21]. Well-aggregated soil is stable enough to resist wind and water erosion, and has better air and water infiltration rates favorable for plant and microbial growth [22, 23]. Additionally, GRSP is recalcitrant enough to have a long residence time in soils, and plays a pivotal role in long-term carbon/nitrogen storage and HM sequestration [19, 24]. Therefore, the release and accumulation of GRSP in soils can be a very important mechanism for ecological restoration of soils degraded by mining and smelting activities.

Although the studies concerning the responsiveness of GRSP for agricultural and land-use practices are increasing [25], we still have a poor understanding of the potential changes and distribution characteristics of GRSP at different soil depths in HM contaminated soils. GRSP is a glycoprotein produced by mycelium and spore walls of AMF, presenting correlation with mycorrhizal colonization, spore density, hyphal length and other soil properties to some extent [26]. Our previous study reported that long-term HM pollution had significant effects on AMF status and soil properties [27], thereby probably disturbing GRSP production and aggregate formation. We also found that *Sophora viciifolia* was a dominant plant species grown widely in HM polluted soils and commonly colonized by AMF [28]. As pioneer plant species, they appeared to be suitable for phytoremediation and revegetation of HM contaminated sites due to their fast growth, deep root system and high stress resistance [28, 29]. However, a detailed information about the effects of AMF on distribution of GRSP, aggregate stability and soil quality at different soil depths in HM polluted area are still lacking. Therefore, better understandings of GRSP distribution, aggregate composition, AMF status, soil properties and their relationships along gradients of HM contamination is required for phytoremediation improvement and degraded ecosystem restoration. The present study was conducted to (1) evaluate the influences of HM contamination on soil properties, AMF status, GRSP and aggregate distribution at different soil depths; (2) analyze the relationships among these measured index; and (3) identify the important biological factors affecting aggregate-size distribution and stability in HM contaminated area.

## Materials and methods

### Ethics statement

The study area is not privately-owned or protected in any way, so no specific permits were required for the described field studies. The field studies did not involve endangered or protected species. Informed consent was obtained from all participants.

### Study area and sampling

The study was conducted during the wet season in September 2011 in Qiandongshan lead (Pb) and zinc (Zn) polluted area which is located about 230 km to the west of Xi’an city (the capital of Shaanxi Province). The area is one of the four largest Pb and Zn bases in China with an annual production capacity of 30,000 tons of Pb concentrates, 100,000 tons of Zn concentrates, 10,000 tons of electrolytic Pb, and 5,000 tons of Pb alloy. The mineral resources are very abundant in this area and mining industry has become an important economic mainstay in Shaanxi Province. In addition, the Qiandongshan Pb and Zn polluted area is the largest and the most typical of five national nonferrous metal planning mines, accounting for 25% of the total reserves of Feng County [30]. This area was operated on a small scale from 1985 until it became a large-scale operation in 1996 [28]. The predominant pollution sources in this region are mine wastewater, beneficiation wastewater, and mine tailings.

The study area belongs to a warm temperate semiarid climate, with maximum temperature of 22.7°C, minimum temperature of -1.1°C and average annual temperature of 11.4°C. The annual average rainfall and frost-free period are 613.2 mm and 188 days, respectively. It has an elevation ranging from 1,000–2,200 m above sea level, and the soil type is cinnamon and brunisolic and the soil texture is from light to heavy according to the traditional soil genesis classification in China [31].

Five sampling sites in this area with gradually increasing concentrations of HM were selected according to our previous study [4, 28, 29]: S1, S2, S3, S4, located within 50, 150, 500, 1500 m to the west of a smelter and mine (33°51'17"N, 106°39'25"E), respectively (S1 Fig). The S5 (33°48'03"N, 106°46'26"E), intended as a control site, was located approximately 12 km to the southeast of the smelter and mine, where no HM pollution was known to exist. The division of polluted soils into different levels was based on the variations of Pb and Zn concentrations and recommendation of environmental quality standard (Grade II) in soils of China (GB 15618–1995).

Six sampling plots at each site were selected on the basis of having dominated plant (*S*. *viciifolia*) [28, 29], and an individual of dominated plant was randomly selected from each sampling plot (3 × 3 m). After removing the surface residue (5 mm), the undisturbed soil blocks (10 cm length × 5 cm width × 10 cm height) from 0–10, 10–20, 20–30 and 30–40 cm soil depths were taken with a clean spade, knife, and trowel directly beneath the patches of dominated plant. One portion of soil blocks (5 cm length × 5 cm width × 10 cm height) collected were stored in plastic boxes and transported to laboratory for soil physical and chemical, AMF spore density (SP) and hyphal length density (HLD) assay, while the other portion of soil blocks (5 cm length × 5 cm width × 10 cm height) were passed through a 1 mm sieve to obtain sufficient roots samples of *S*. *viciifolia* for mycorrhizal colonization (MC) analysis.

### Soil chemical properties

Portions of soil samples were dried by spreading them out on paper in ambient air and at room temperature for two weeks, and then were homogenized, grounded in an agate mortar and sieved to 0.2 mm for soil chemical property analysis. Soil pH was determined in a suspension with deionized water (soil/water, 1:5) using a pHS-3D digital pH meter (Leici, Shanghai, China) with a combined glass-calomel electrode. Soil organic matter (SOM) was measured by the wet combustion method using a mixture of potassium dichromate and sulfuric acid under heating [32]. Soil organic carbon (SOC) concentration was determined by dry combustion method using total organic carbon analyzer (TOC-VCPH, Shimadzu, Japan). Total nitrogen (TN) concentration was determined according to the semi-micro Kjeldahl method [33]. Total phosphorus (TP) concentration was measured colorimetrically after wet digestion with HF-HClO_4_ according to Jackson [34]. For the assessment of total Pb and Zn concentrations, 0.5 g soil material was accurately weighed and digested with a mixture of HNO_3_ and HCl (aqua regia digestion) in a ratio 3:1 (v/v). The extractable Pb and Zn concentrations were determined by treating 2 g soil material with DTPA solution (0.005 M diethylene triamine penta-acetic acid (DTPA), 0.01 M CaCl_2_, 0.1 M triethanolamine, pH = 7.3). The digests were then analyzed for metal concentrations using flame atomic absorption spectrometry (FAAS, Hitachi Z-2000, Tokyo, Japan). The blank reagent and standard reference soil were assayed for quality assurance and quality control [4].

### Mycorrhizal colonization

For the evaluation of mycorrhizal colonization (MC), root samples were taken out from FAA, washed several times with running tap water, and cut into 1-cm length segments which were then stained according to the modified method of Koske and Gemma [35]. The root segments were first softened with 5% KOH at 90°C in a water bath for 40 min, bleached with fresh alkaline H_2_O_2_ at room temperature for 30 min, acidified with 2% HCl for 10 min, and then stained in 0.05% (w/v) trypan blue solution (200 mL phenol, 0.5 g trypan blue, 250 mL lactic acid, 250 mL glycerol, and 300 mL distilled water) at room temperature for 2 h. The stained root samples were then transferred into acidified glycerol and incubated for 12 h at room temperature. The MC was estimated according to the method modified by Trouvelot et al [36]. More than one hundred root segments per specimen were used to measure the MC under a light microscope (Olympus BX51, Japan) at 200× magnification. The percentage of mycorrhizal structures in each 1 cm root fragment was assessed as 0, 10, 20 … 100%. The intensity of MC was calculated using the following equation [37]:

$$\text{MC }\left(\text{\%}\right)=\frac{\sum \left(0\%\times N_{0}+10\%\times N_{10}+20\%\times N_{20}+\cdots +100\%\times N_{100}\right)}{\left(N_{0}+N_{10}+N_{20}+\cdots +N_{100}\right)}$$

Where *N* is the number of root fragments.

### AMF spore density

AMF spores were isolated from soil samples using wet sieving method described by Daniels and Skipper [38]. Briefly, fifty grams of soil from each plant rhizosphere was independently suspended in 500 mL water, stirred with a glass stirring rods for 0.5 min, and the suspension passed through a sequence of sieves (500, 250, 100 and 30 μm). Spores were collected from the last two sieves with tap water, and then the residues from the sieves were filtered through filter paper using a vacuum pump. The filter paper was placed on a 9-cm Petri dish, and the number of AMF spores was counted under a binocular stereomicroscope using a hand tally counter. Spores were counted and categorized as live and dead: dead spores typically lacking cytoplasm, and/or showing signs of parasitism [39]. AMF spore density (SP) was expressed as number of spores in 1 g of dry soil.

### Hyphal length density of AMF

Hyphal length density (HLD) was measured by the modified grid-line intersection method described by Jakobsen et al [40]. A soil subsample (5 g) was blended for 20 s with distilled water. The suspension was then poured through a 250 μm and a 32 μm sieve to separate the hyphae. The material was suspended again in 250 mL distilled water, transferred to a beaker, shaken for 30 s, and then left to settle for 5 min at room temperature. The supernatant (containing hyphae) was filtered on to the filtration apparatus under vacuum. The procedure was repeated 3 times on each subsample for thorough extraction of soil fungal hyphae. The hyphae recovered from the six subsamples taken from each site were stained by a 0.05% (w/v) trypan blue solution. The hyphal length was measured by the gridline intercept method at 200× magnification under a microscope and the HLD was presented in units of m g^-1^ dry soil.

### Soil GRSP concentration

The concentrations of easily extractable glomalin-related soil protein (EE-GRSP) and total glomalin-related soil protein (T-GRSP) were measured according to procedures described by Wright and Upadhyaya [41]. Briefly, 1 g soil sample was placed into a centrifuge tube. The EE-GRSP was incubated with 8 mL of 20 mM citrate solution (pH 7.0), autoclaved at 121°C and 0.11 Mpa for 30 min, and then centrifuged at 10,000 g for 5 min to remove residual soil particles. The T-GRSP was extracted with 8 mL of 50 mM citrate solution (pH 8.0) by autoclaving at 121°C for 60 min. After each autoclaving cycle, the supernatant was removed by centrifugation at 5,000 g for 20 min for sequential extraction. The extraction of a soil sample continued until the supernatant showed none of the red-brown color. After extraction cycles were completed, extracts from each replicate were pooled and then centrifuged at 10,000 g for 5 min to remove residual soil particles. Protein in the supernatant was determined by the Bradford dye-binding assay with bovine serum albumin as a standard [41].

### Aggregate size distribution

The concentration of water stable aggregates (WSA) was measured and the four soil aggregate fractions were separated according to the wet-sieving method [42]. Briefly, a series of three sieves was used to collect four aggregate size fractions: (1) >2000 μm (large macroaggregates); (2) 2000–250 μm (small macroaggregates); (3) 250–53 μm (microaggregates); (4) <53 μm (silt and clay fraction). Fifty grams of air-dried soil was prewetted by submerging with distilled water overnight at room temperature to equilibrate. During sieving, aggregates were separated by manually moving the sieve up and down 3 cm with 50 repetitions during a period of 2 min. Finally, the residue on each sieve were then collected over a preweighed filter paper, oven dried at 60°C for 48 h until steady weight was achieved. The water stable aggregates (WSA) fraction was expressed as a percentage of WSA size against total dry soil sample. Aggregate stability was measured as the mean weight diameter (MWD) of stable aggregates as equation:

$$MWD\;\left(\%\right)=\sum_{i=1}^{n+1}\frac{r_{i-1}+r_{i}}{2}\times m_{i}$$

where *r*_0_ = *r*_1_, *r*_1_ = 2 mm, *r*_2_ = 0.25 mm, *r*_3_ = 0.053 mm, *r*_3_ = 0 mm, *m*_*i*_ is the proportion of soil aggregates on the *i*^*th*^ sieve, and *n* is the number of the sieves.

### Chlorophyll concentration and net photosynthetic rate of *S*. *viciifolia*

The terminal leaflet of the 4^th^, fully expanded, compound leaf (from the top) of each *S*. *viciifolia* grown at different study sites was used to determine chlorophyll (Chl) concentration and net photosynthetic rate (Pn). The Chl concentration was measured by using a SPAD Chl meter (SPAD-502, Konica Minolta Sensing, Inc., Japan) according to the manufacturer's instruction. The Pn was determined by using a portable open flow gas-exchange system LI-6400 (LI-6400, LI-COR, Lincoln, NE, USA) from 08:30 to 11:30 in the morning. The photosynthetically active irradiation was 1200 μmol m^–2^ s^–1^, CO_2_ concentration was 400 cm^3^ m^–3^, the leaf temperature was 25°C, and the air flow rate was about 0.5 dm^3^ min^–1^.

### Statistical analysis

Prior to data analysis, the Kolmogorov-Smirnov test was used to check for data normality and the Levene test for homogeneity of variance in SPSS 16.0 for Windows 7 (SPSS Inc., Chicago, IL, USA). In the present study, all the original datasets conformed to a normal distribution. When necessary, dependent variables were transformed using the natural logarithmic, arcsine or Box-Cox functions to achieve requirements of homogeneity of variance (*P* > 0.05). Potential differences in parameters among study sites and plant species were analyzed using one-way followed by Student-Newman-Keuls (SNK) test. One-way ANOVA was used to determine the differences among soil properties, HM concentrations, MC, SP, GRSP and Chl concentrations, Pn, WSA and MWD at different soil depths. Pearson correlation coefficients were used to analyze the relationships among soil properties, HM concentrations, MC, SP, GRSP and Chl concentrations, Pn, WSA and MWD. All tests were two-tailed and significance of the obtained results was judged at the 5% level. Redundancy analyses (RDA) were conducted to determine the multivariate relationship among soil aggregate distribution, stability and soil properties at different soil depths using the software Canoco (version 4.5, Centre for Biometry, Wageningen, The Netherlands). All data in the figures and tables are presented with original data, and they are presented as mean ± SD (standard deviation).

## Results

### Soil chemical properties and HM concentrations

The chemical properties and HM concentrations in the rhizosphere soil of *S*. *viciifolia* grown at five different study sites are presented in Table 1. Soil pH was variable, ranging from neutral to slightly alkaline or alkaline values (6.85–8.79). The highest SOM concentration (12.7 mg g^-1^) was found at S3 (10–20 cm soil depth), while the lowest value (6.01 mg g^-1^) could be detected at S5 (20–30 cm soil depth). A large variability was found with respect to SOC, ranging from 10.2–22.7 mg g^-1^, and the highest and the lowest values appeared at S2 (0–10 cm soil depth) and S5 (30–40 cm soil depth) respectively. However, there was less variability in TN (0.96–1.62 mg g^-1^) and TP concentrations (0.92–1.63 mg g^-1^) than other soil properties. The largest and the lowest values of TN concentrations were found at S2 (10–20 cm soil depth) and S4 (20–30 cm soil depth), respectively. S3 (0–10 cm soil depth) had the highest TP concentration, while the lowest TP concentration could be detected at S5 (30–40 cm soil depth).

**Table 1:** Soil chemical properties at different soil depths and study sites.

Sites	Depth (cm)	pH	SOM (mg g^-1^)	SOC (mg g^-1^)	TN (mg g^-1^)	TP (mg g^-1^)
S1	0–10	8.14±0.82a	9.93±1.71a	18.6±3.20a	1.32±0.15a	1.31±0.20a
	10–20	8.21±1.27a	11.3±1.87ab	17.2±2.04ab	1.45±0.24a	1.27±0.14a
	20–30	8.79±0.29a	9.12±1.26ab	13.2±3.18b	1.08±0.20b	1.04±0.23b
	30–40	8.28±0.27a	7.96±1.44b	13.7±3.13b	1.02±0.18b	0.93±0.13b
S2	0–10	8.35±0.54a	12.3±1.63a	22.7±3.13a	1.39±0.32ab	1.47±0.28a
	10–20	8.29±0.87a	12.4±1.79a	18.7±3.81ab	1.62±0.21a	1.49±0.25a
	20–30	8.18±0.50a	9.36±1.76b	15.8±3.24b	1.06±0.18b	1.13±0.18b
	30–40	8.50±1.27a	10.4±1.82ab	15.7±3.60b	1.21±0.18b	1.02±0.18b
S3	0–10	8.33±0.55a	10.9±1.19a	19.5±2.41a	1.57±0.34a	1.63±0.29a
	10–20	8.20±0.41a	12.7±1.73ab	21.1±2.19a	1.43±0.28ab	1.24±0.20b
	20–30	8.54±0.78a	10.9±1.94ab	15.6±1.74ab	1.30±0.06ab	1.28±0.20b
	30–40	8.13±1.08a	9.27±2.21b	17.7±3.42b	1.17±0.16b	1.14±0.16b
S4	0–10	8.30±0.70a	10.1±2.00a	16.6±2.41ab	1.37±0.31a	1.29±0.09ab
	10–20	7.95±0.30a	7.59±2.16ab	19.6±3.34a	1.34±0.09a	1.47±0.36a
	20–30	8.35±0.78a	6.48±1.05ab	15.0±2.48b	0.96±0.17b	1.02±0.19b
	30–40	8.10±0.12a	7.77±1.77b	14.2±3.36b	1.05±0.19b	0.99±0.18b
S5	0–10	7.09±0.66ab	6.14±1.37b	11.4±1.25a	1.00±0.15a	1.09±0.23a
	10–20	6.85±0.55b	7.15±1.17ab	12.7±2.48a	1.18±0.14a	1.17±0.14a
	20–30	7.34±0.35ab	6.01±1.65b	11.9±2.80a	1.05±0.11a	1.00±0.10a
	30–40	7.79±0.55a	8.17±1.10a	10.2±1.40a	1.02±0.18a	0.92±0.18a

SOM, soil organic matter; SOC, soil organic carbon; TN, total nitrogen; TP, total phosphorus. Each value is the mean (±SD) of six replicates (SNK test, *P* < 0.05). Different letter indicates statistically significant difference (one-way ANOVA, *P* < 0.05) at four soil depths (0–10, 10–20, 20–30, 30–40 cm).

**Fig 1:**
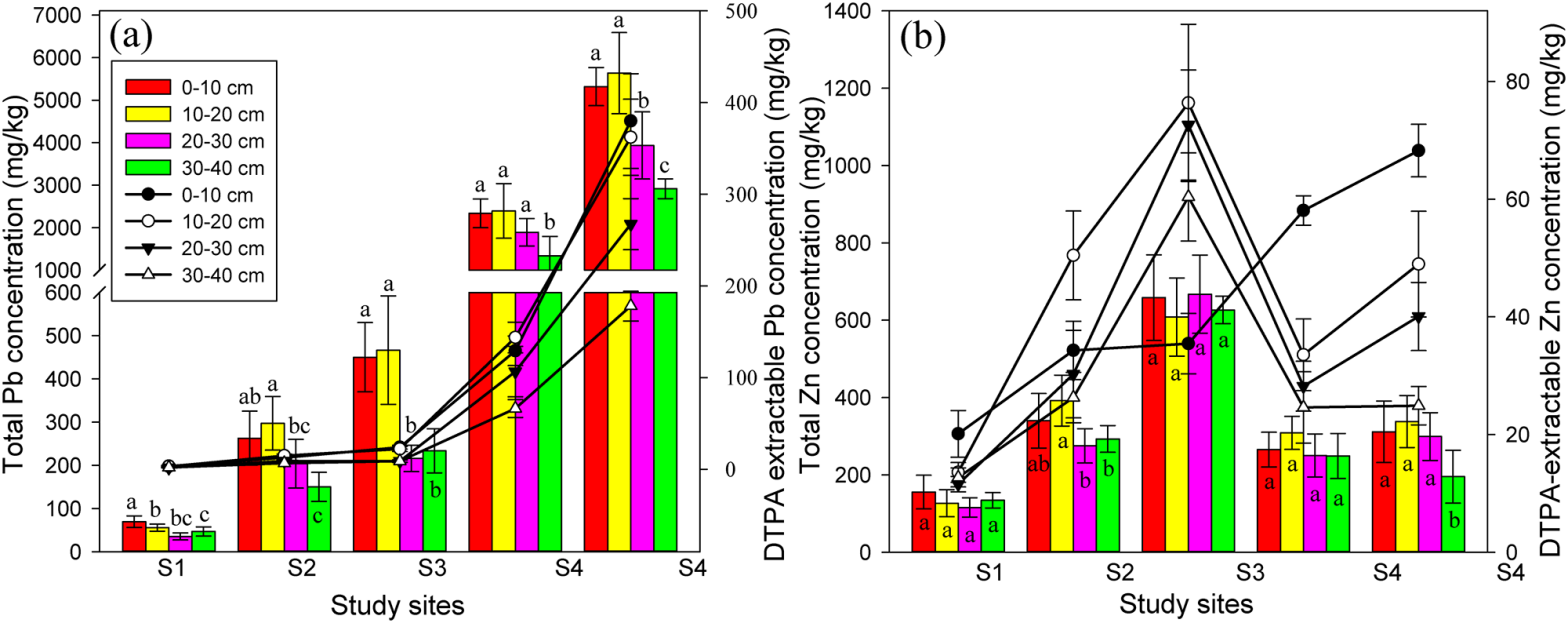
Soil total/DTPA-extractable Pb (a) and Zn (b) concentrations at different soil depths and study sites. The bar charts represent total Pb or Zn concentration, while the line graphs show DTPA-extractable Pb or Zn concentration. Each value is the mean ± SD (n = 6). Different letters indicate statistically significant differences (one-way ANOVA followed by SNK test, *P* < 0.05) at four soil depths (0–10, 10–20, 20–30, 30–40 cm).

The rhizosphere soil of *S*. *viciifolia* varied greatly in total/DTPA-extractable Pb and Zn concentrations at different soil depths and studied sites, ranging from 35.4–5636 mg kg^-1^ for total Pb, 116–668 mg kg^-1^ for total Zn, 1.66–380 mg kg^-1^ for DTPA-extractable Pb, and 11.6–76.4 mg kg^-1^ for DTPA-extractable Zn (Fig 1, S1 Table). The concentrations of DTPA-extractable HM in relation to total concentrations were 5.37% for Pb and 11.61% for Zn when all soil samples from different depths and study sites were taken into consideration. There were significantly positive correlations between DTPA-extractable Pb and total Pb (*P* = 0.000), and DTPA-extractable Zn and total Zn (*P* = 0.000). Total/DTPA-extractable Pb also had notably positive relationship with Pb and Zn availabilities (*P* < 0.05) except for soil samples at 10–20 cm depth (S2 Table).

### Mycorrhizal colonization, AMF spore density and hyphal length density

**Fig 2:**
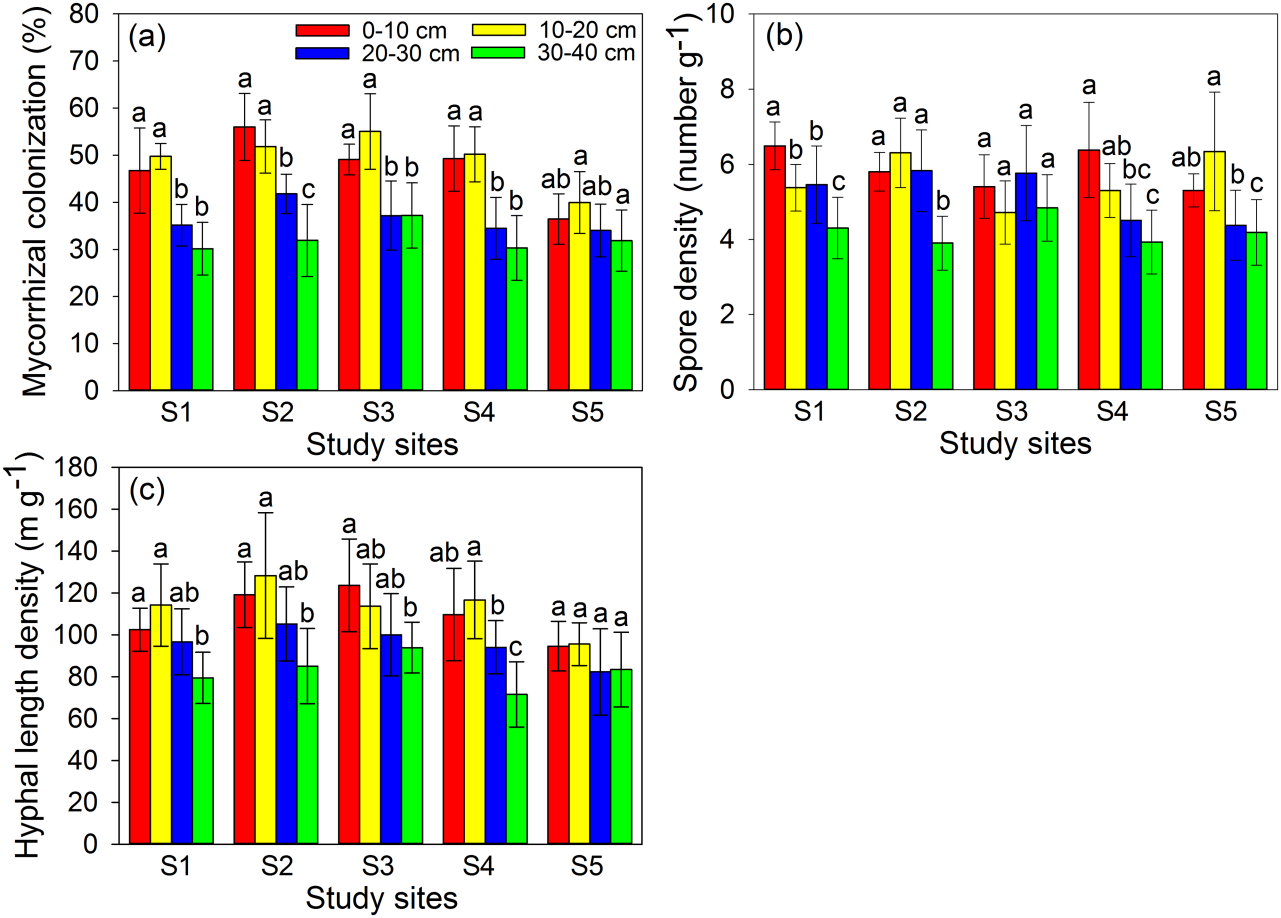
Mycorrhizal colonization (MC, a), AMF spore density (SP, b) and hyphal length density (HLD, c) at different soil depths and study sites. Each value is the mean ± SD (n = 6). Different letters indicate statistically significant differences (one-way ANOVA followed by SNK test, *P* < 0.05) at four soil depths (0–10, 10–20, 20–30, 30–40 cm).

Mycorrhizal colonization (MC) in plant roots, spores and hyphae of AMF in rhizosphere soil of *S*. *viciifolia* could be found even at the most heavily contaminated site (S2 Fig). The MC, spore density (SP) and hyphal length density (HLD) of AMF varied among different soil depths and study sites, ranging from 30.2–56.0% for MC, 3.91–6.49 number g^-1^ for SP and 71.5–128 m g^-1^ for HLD (Fig 2). The root and soil samples at 0–10 cm soil depth had much higher MC, SP and HLD compared with that at 30–40 cm soil depth. There were significantly positive correlations between MC and SOM, SOC, but negative correlations could be found between MC and total/DTPA-extractable Pb at 0–10 and 10–20 cm soil depths (S3 and S4 Tables). However, no correlations could be found between MC and soil properties at 20–30 and 30–40 cm soil depths (*P* > 0.05). Similarly, soil properties did not have significant influence on SP in all soil samples except for SOM at 20–30 cm soil depth (*P* < 0.01). HLD had significantly positive correlations with SOM, SOC, TN and TP at 0–10 cm soil depth, but was greatly inhibited by total/DTPA-extractable Pb at 0–10, 10–20 and 20–30 cm soil depths.

### GRSP concentration

**Fig 3:**
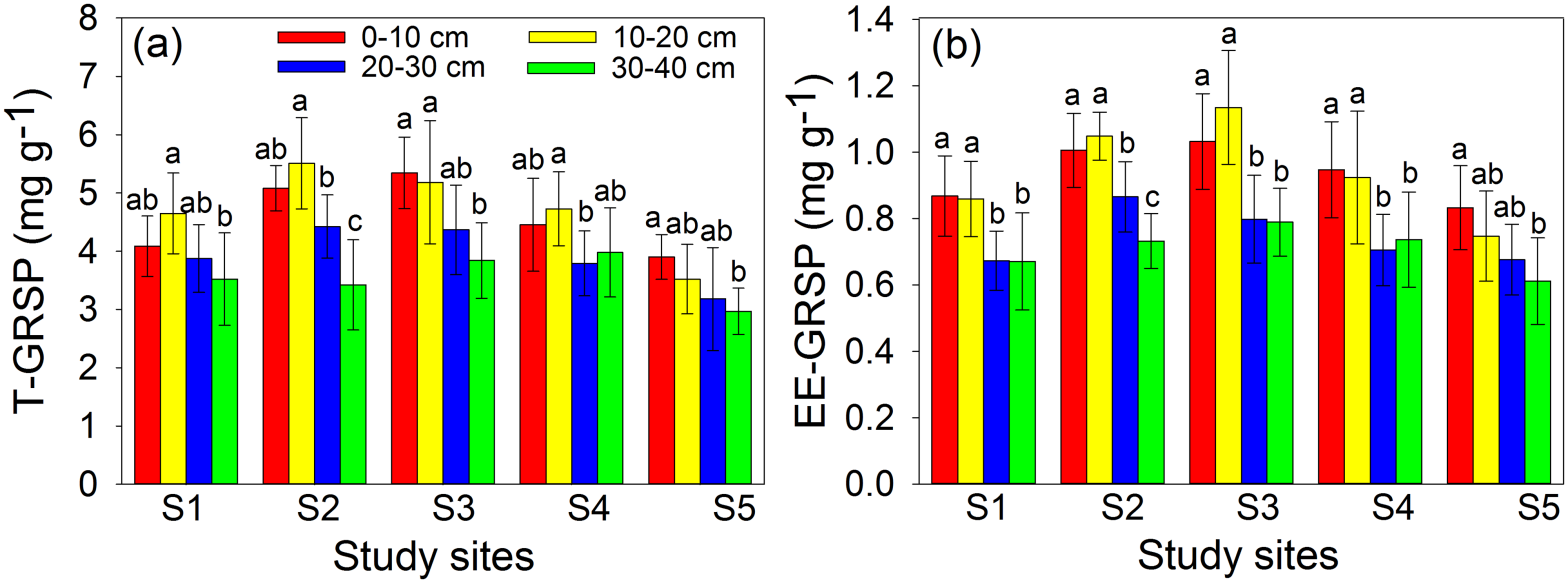
Total glomalin-related soil protein (T-GRSP, a) and easily extractable glomalin-related soil protein (EE-GRSP, b) concentrations at different soil depths and study sites. Each value is the mean ± SD (n = 6). Different letters indicate statistically significant differences (one-way ANOVA followed by SNK test, *P* < 0.05) at four soil depths (0–10, 10–20, 20–30, 30–40 cm).

The concentrations of T-GRSP and EE-GRSP in rhizosphere soil of *S*. *viciifolia* at different soil depths and study sites decreased with increase of HM contamination level, varying from 2.97–5.51 mg g^-1^ for T-GRSP and 0.61–1.13 mg g^-1^ EE-GRSP, respectively (Fig 3). T-GRSP was significantly and positively correlated with SOM, SOC and TN, but negatively correlated with total/DTPA-extractable Pb and Pb availability at 0–10, 10–20 and 20–30 cm soil depths (S2 and S3 Tables). EE-GRSP had significantly positive correlation with SOC at all soil depths (*P* < 0.05), but positive correlated with pH, SOM and TN only at 10–20 cm soil depth (*P* < 0.05). Additionally, the significantly negative correlation could be found between EE-GRSP and total/DTPA-extractable Pb at both 0–10 and 10–20 cm soil depths (*P* < 0.05).

### Soil aggregate distribution and MWD

**Fig 4:**
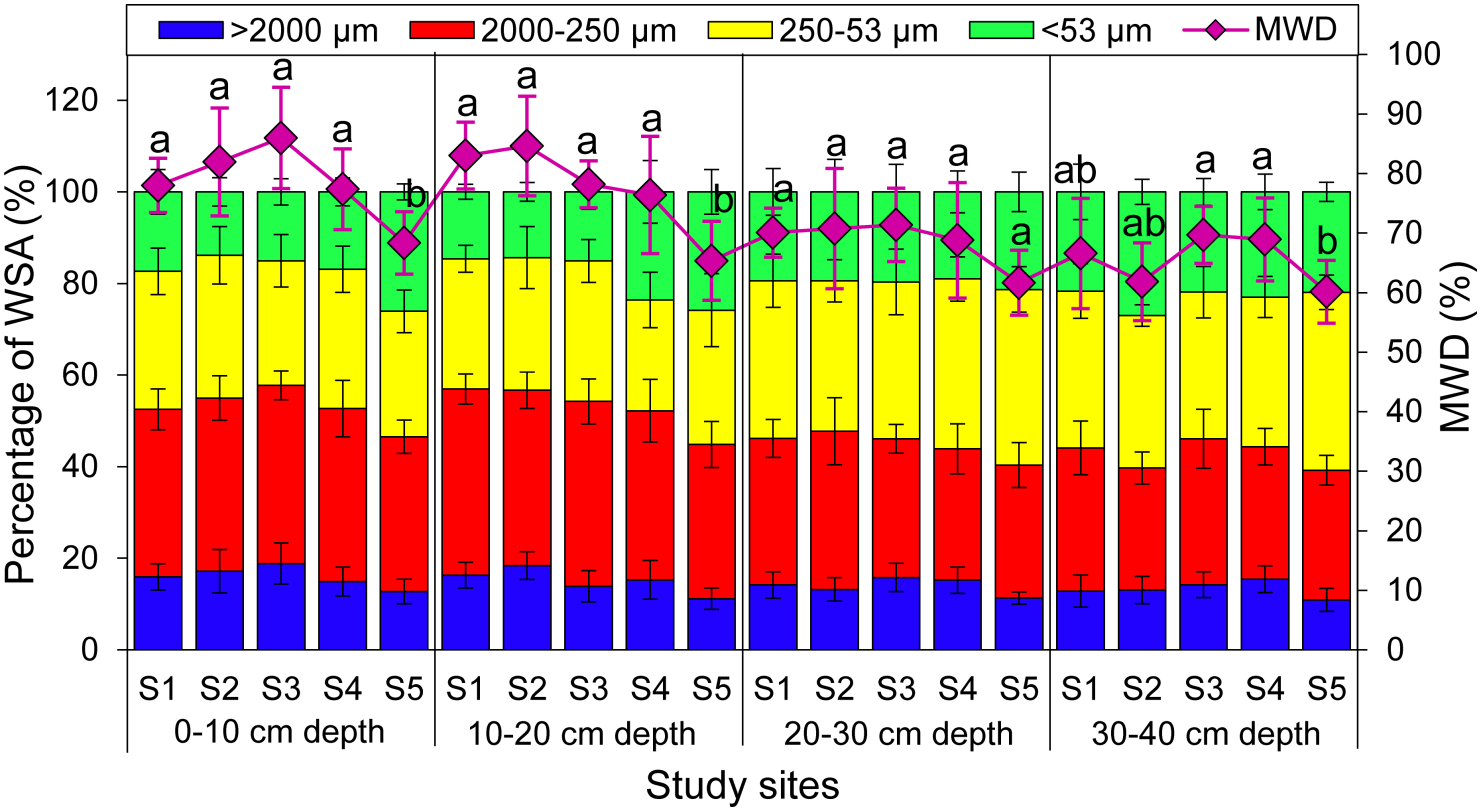
The percentage of water-stable aggregates (WSA, bar chart) and mean weight diameter (MWD, line graph) of rhizosphere soils at different soil depths and study sites. Each value is the mean ± SD (n = 6). Different letters indicate statistically significant differences (one-way ANOVA followed by SNK test, *P* < 0.05) of MWD at five study sites (S1, S2, S3, S4 and S5).

Aggregate-size distribution was dominated by small macroaggregates (2000–250 μm) and microaggregates (250–53 μm) in all soil samples, totally accounting for 59.9–71.0% of the dry soil weight followed by silt and clay (<53 μm) with 13.9–27.0%. However, large macroaggregates (>2000 μm) made up the lowest proportion, ranging from 10.9–18.9% (Fig 4). Soil samples at 0–10 cm depth had significantly higher percentage of WSA_2000-250 μm_ but lower percentage of WSA_<53 μm_ compared with that at 30–40 cm depth.

The mean weight diameter (MWD) of soil aggregates exhibited a high variability (0.62–0.86) at different soil depths and study sites (Fig 4). MWD decreased with increase of soil HM concentration, and the MWD at top soil was significantly higher than that at bottom soil at all study sites (*P* < 0.05).

### Correlations among GRSP concentration, soil properties, AMF status, and aggregate distribution

T-GRSP had significantly positive correlations with SOM, SOC and TN (*P* < 0.05), but was negatively correlated with total/DTPA-extractable Pb at 0–10, 10–20 and 20–30 cm soil depths (Table 2). However, no correlation could be detected between T-GRSP and total/DTPA-extractable Pb at 30–40 cm soil depth (*P* > 0.05). EE-GRSP was significantly and positively correlated with SOC at all soil depths, while had significantly negative correlations with total/DTPA-extractable Pb at only shallow soil layer (0–20 cm).

**Table 2:** Correlational analysis among GRSP concentration, soil properties AMF status at different soil depths and study sites.

Soil properties	T-GRSP				EE-GRSP			
	0–10 cm	10–20 cm	20–30 cm	30–40 cm	0–10 cm	10–20 cm	20–30 cm	30–40 cm
pH	0.30NS	0.42*	0.34NS	0.52**	0.28NS	0.42*	-0.02NS	0.11NS
SOM	0.43*	0.50**	0.56**	0.08NS	0.30NS	0.52**	0.47**	-0.07NS
SOC	0.53**	0.53**	0.48**	0.61**	0.43*	0.57**	0.46*	0.39*
TN	0.47**	0.47**	0.40*	0.20NS	0.25NS	0.44*	0.18NS	0.03NS
TP	0.36NS	0.25NS	0.41*	0.34NS	0.32NS	0.33NS	0.37*	0.57**
TPb	-0.42*	-0.60**	-0.53**	-0.22NS	-0.38*	-0.42*	-0.32NS	-0.31NS
DPb	-0.46*	-0.61**	-0.52**	-0.30NS	-0.36*	-0.49**	-0.32NS	-0.36NS
TZn	0.51**	0.24NS	0.32NS	0.20NS	0.32NS	0.41*	0.38*	0.32NS
DZn	0.51**	0.16NS	0.17NS	0.18NS	0.29NS	0.28NS	0.26NS	0.19NS
MC	0.42*	0.38*	0.15NS	-0.02NS	0.41*	0.54**	0.16NS	-0.02NS
SP	-0.23NS	-0.10NS	0.40*	0.15NS	-0.18NS	-0.31NS	0.52**	0.02NS
HLD	0.68**	0.41*	0.42*	-0.34NS	0.40*	0.43*	0.16NS	-0.04NS

SOM, soil organic matter; SOC, soil organic carbon; TN, total nitrogen; TP, total phosphorus; TPb, total Pb; TZn, total Zn; DPb, DTPA-extractable Pb; DZn, DTPA-extractable Zn; MC, mycorrhizal colonization; SP, spore density; HLD, hyphal length density.

**P < 0.01;

*P < 0.05;

NS, not significant.

Soil EE-GRSP, pH, SOM and SOC had significantly positive correlations with WSA_>2000 μm_ and MWD, but were negatively correlated with WSA_<53 μm_ at 0–10 cm depth. However, both total and DTPA-extractable Pb showed significantly negative correlations with WSA_>2000 μm_ and MWD at 0–30 cm depth (S5 Table). Soil T-GRSP and EE-GRSP were significantly and positively correlated with WSA_>2000 μm_ and MWD at 10–20 cm depth, but no significant correlations could be found between T-GRSP, EE-GRSP and WSA_2000-250 μm_, WSA_250-53 μm_ and WSA_<53 μm_ at 10–20 and 30–40 cm depths. In addition, MC had significantly positive relationship with MWD at 0–20 cm depth, while SP was significantly and positively correlated with WSA_>2000 μm_, WSA_2000-250 μm_ and MWD at 20–30 cm depth (S5 Table).

## Discussion

### HM Pollution levels at different study sites

The maximum total concentrations of Pb and Zn at S1 and S2 did not exceed their corresponding limits of the National Soil Environmental Quality Standard II (GB15618-1995), indicating that the HM concentrations at S1 and S2 were normal (Fig 1). However, the total concentrations of Pb at S4, S5 and Zn at S3 were 5.69, 12.72 and 2.13 times above grade II of GB15618-1995 respectively, suggesting that these sites were severely contaminated by Pb or Zn according to either national grade II standard or Shaanxi provincial background values. The results reflected that long-term smelting and mining activities could result in significant accumulations of Pb and Zn in soils. The slope of the linear regression line with the order of Zn>Pb indicated the higher solubility and availability of Zn compared with that of Pb in soils, which was consistent with results of other researchers for contaminated soils around mining area [43].

### HM availability and correlations with soil properties

It has been accepted that HM speciation and the resulting availability rather than total HM concentration determines the overall physiological and toxic effects of a metal on biological systems [44]. In this study, the availability of HM was defined as the ratio of the DTPA-extractable concentration to the corresponding total concentration of HM to characterize HM toxicity. The availability of Zn (11.61%) was significantly larger than that of Pb (5.37%), indicating that Zn had higher mortality than Pb when present at the same concentration (S3 Fig). In addition, soil pH and SOM are two of the most important soil properties influencing the speciation, movement, and final or actual HM availability [45]. Our study showed that the availabilities of Pb and Zn had significantly negative correlations with soil pH (Pb: *r* = -0.374, *P* = 0.000; Zn: *r* = -0.402, *P* = 0.000) and SOM (Pb: *r* = -0.329, *P* = 0.000; Zn: *r* = -0.226, *P* = 0.013) when all soil samples were taken into account (S4 Fig). Horckmans et al [46] revealed that a decline in soil pH could increase HM availability as a result of proton competition with the metals and reduction in negative binding sites. The results were also in agreement with Liu et al [47] who reported that SOM could reduce HM availability by forming stable complexes.

### AMF status and correlations with soil properties

Arbuscular mycorrhizal fungi (AMF) are the most important soil microorganisms, which can form universal symbiosis with more than 80% of terrestrial plants [9]. However, both biotic and abiotic factors have been shown to influence the AMF diversity, mycorrhizal colonization, hyphal growth and spore production [21, 48]. In the current study, both MC and HLD had significantly positive correlations with SOM, SOC and TP at 0–10 cm soil depth (*P* < 0.05), but were significantly inhibited by total/DTPA-extractable Pb at 0–10 and 10–20 cm soil depths (*P* < 0.05). SP was not correlated with soil properties except for SOM at 20–30 cm depth (*P* < 0.01) and was significantly inhibited by total/DTPA-extractable Pb at 20–30 cm soil depth (*P* < 0.01). Our finding was consistent with Rillig et al [49] who presented evidence that AMF could make large, direct contributions to SOM and carbon sink, which highlighted the importance of AMF in ecological restoration. The results were also consistent with our previous studies which reported that the MC were significantly lower in HM contaminated soils [27, 28]. Wu et al [50] indicated that elevated concentrations of As, Pb, Zn, Cd and Cu exerted harmful effects on MC through inhibiting spore production and fungal spread in abandoned As/Pb/Zn mines. However, AMF propagules never disappeared completely even in soils contaminated by high concentrations of HMs (S2 Fig), suggesting that some species of AMF might have developed a certain degree of stress adaptation.

### GRSP concentration and correlations with soil properties

Glomalin is produced by living hyphae of obligate biotrophic AMF and the concentration depends on soil properties, climate, fungi involved, the host plants and their productivity [49]. In the present study, T-GRSP and EE-GRSP had significantly positive relationships with SOM, SOC and TN concentrations when all soil samples were taken into consideration (*P* < 0.01), suggesting that GRSP, SOM, SOC and TN concentrations are probably subjected to similar deposition and decomposition dynamics (S3 Table). The highly significant correlation between SOM and GRSP, again confirmed that glomalin was a significant component of the organic matter complex [51]. Our findings were consistent with Rillig et al [52] who demonstrated that glomalin was a significant component of the soil C and N pools, and accounted for about 27% of the sources of SOM. However, in the current study, there were significantly negative correlations between T-GRSP/EE-GRSP and total/DTPA-extractable Pb at 0–10 and 10–20 cm soil depths (S4 Table), indicating that HM exhibited toxic effects on GRSP accumulation. The negative influence of HM on GRSP production could be partly explained by the phenomenon that the net photosynthetic rate of host was greatly inhibited by HM pollution (S5 Fig), resulting in low carbon deposit into the rhizosphere soil and thereby retarding the growth of AMF. In addition, T-GRSP and EE-GRSP concentrations were significantly inhibited by Pb compared to Zn at 0–20 cm soil depth, indicating that Pb had greater toxic effects on GRSP accumulation than that of Zn at shallow layer of soil (0–20 cm). Supporting our results, previous study showed that Zn was one of the essential elements for plant and involved in regulating several stages of plant growth and development [53], while relatively low concentration of Pb could exert adverse effects on plant growth, soil structure and nutrient cycling [54]. These correspond well with a negative correlation between GRSP and Pb concentration, and a positive relationship between GRSP and Zn concentration in rhizosphere soil (S4 Table). Other studies have likewise found positive, negative or neutral correlations between HM concentration and soil T-GRSP/EE-GRSP concentrations [51, 55]. These conflicting findings suggested that the effects of HM on soil GRSP concentration probably depend on other environmental factors, such as AMF species, host plant species, stage of plant development, HM speciation and soil properties.

### GRSP concentration and correlations with AMF status

There is increasing circumstantial evidence accumulating from decomposition studies indicating that GRSP is AMF origin [56]. However, the amount of GRSP in a sample may not be related to the biomass of AMF mycelium, since hyphal turnover is very rapid, while GRSP turnover is much slower [57]. In the present study, we detected that T-GRSP and EE-GRSP had significantly positive correlations with MC, SP and HLD when all soil samples were taken into consideration (Table 2), which was consistent with Wu et al [26] and Rillig et al [58] who reported that AMF status effectively mediated the metabolism of GRSP. Additionally, GRSP, produced by AMF, is able to sequester toxic HM [59], revealing the potential use of AMF for bioremediation of soils contaminated with HMs. Furthermore, MWD had significantly positive correlations with T-GRSP and EE-GRSP at 0–10, 10–20 and 20–30 cm soil depths, suggesting the important role of AMF in aggregate stability in HM contaminated soils. However, both T-GRSP and EE-GRSP were significantly inhibited by total/DTPA-extractable Pb at 10–20 and 20–30 cm soil depths, but no correlations were detected among them and Pb concentration at 30–40 cm soil depth, which could be partly explained by the fact that HMs were mainly accumulated in the top soil and downward leaching of HM was little [60]. Therefore, the correlations among Pb and T-GRSP, EE-GRSP were soil depth dependence.

### Soil aggregate distribution, stability and correlations with soil properties at different soil depths

**Fig 5:**
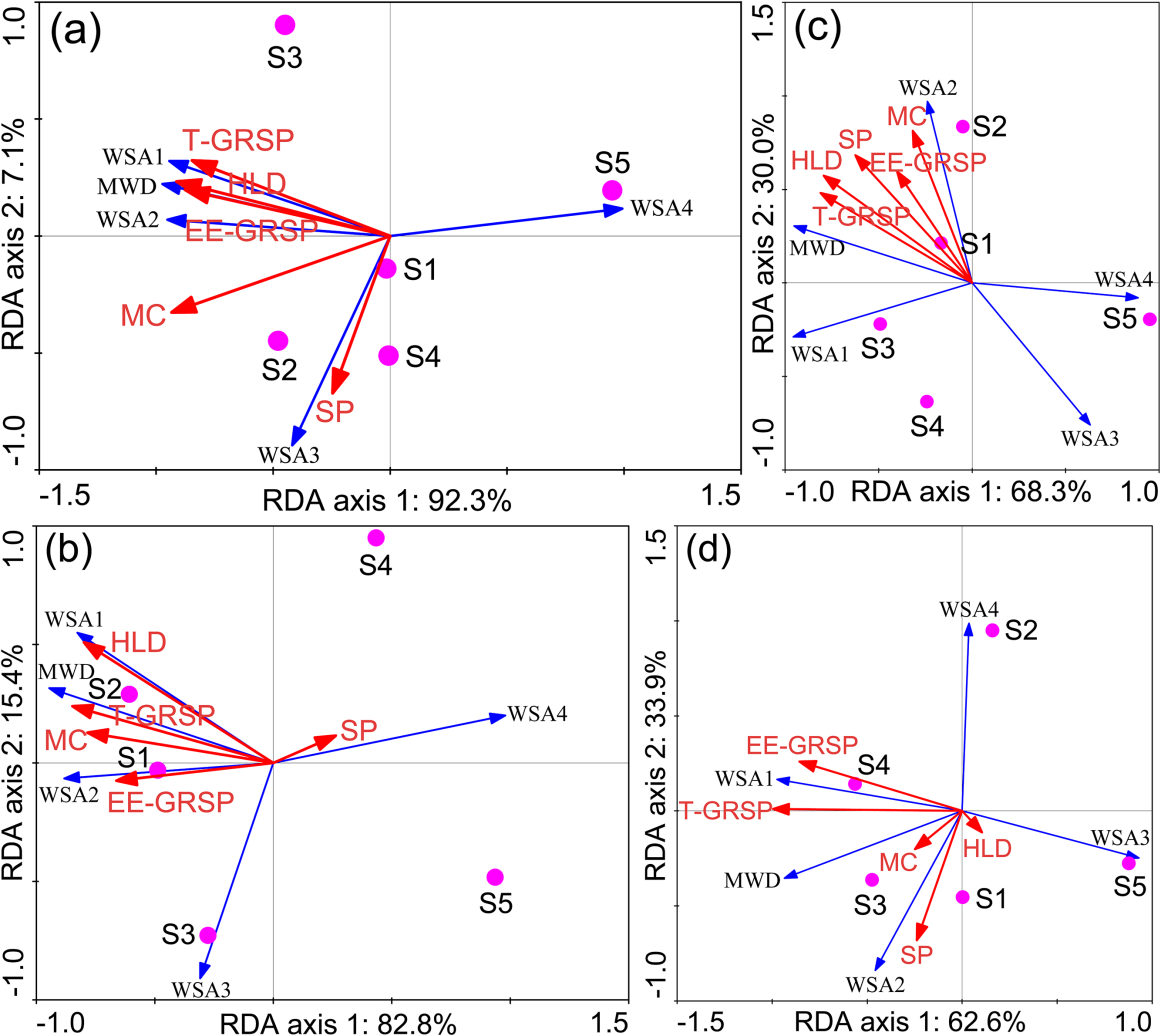
Redundancy analysis (RDA) of the correlations among soil aggregate distribution, stability and soil properties at 0–10 (a), 10–20 (b), 20–30 (c) and 30–40 (d) cm soil depths. WSA1, WSA_>2000 μm_; WSA2, WSA_2000-250 μm_; WSA3, WSA_250-53 μm_; WSA4, WSA_<53 μm_; SOM, soil organic matter; SOC, soil organic carbon; TN, total nitrogen; TPb, total Pb; TZn, total Zn; DPb, DTPA-extractable Pb; DZn, DTPA-extractable Zn.

Numerous studies have indicated that aggregates were essential for storing air and water, microbes, nutrients and organic matter, and the well-aggregated soil is more stable and less susceptible to erosion [61]. In this study, detailed information about aggregate distribution and stability in HM contaminated soils and their influence factors at different depths were analyzed using redundancy analysis (RDA). The length of the red arrows indicates the relative importance of each environmental factor in explaining variation of aggregate distribution and stability, while the angles between the arrows and axis indicate the degree to which they are correlated (Fig 5). At 0–10 cm soil depth, the WSA_>2000 μm_ and MWD had significantly positive correlations with pH, SOM and SOC, while WSA_<53 μm_ was significantly and negatively correlated with pH, SOM, SOC, TN, TP (Fig 5a), suggesting that SOM and SOC were beneficial to large aggregate formation. Furthermore, the improvement in aggregate stability could be partly due to an increase in the proportion of large macroaggregate and decrease in the proportion of silt and clay fraction at 0–10 cm soil depth. WSA_>2000 μm_ and MWD were negatively correlated with total Pb and DTPA-extractable Pb at 0–30 cm soil depth (Fig 5a, 5b and 5c), which was consistent with Vodnik et al [51] who reported that HM and SO_2_ emissions from the smelter were able to cause a serious degradation of the environment (deforestation, soil contamination and erosion). However, no correlations were found among WSA_>2000 μm_, MWD and total/DTPA-extractable Pb at 30–40 cm soil depth (Fig 5d), which could be partly explained by that HMs were mainly accumulated in the top soil layer, with little downward migration [60]. At 20–40 cm soil depth, MWD was positively correlated with either SOM or SOC (S5 Table), indicating that SOM and SOC played an important role in soil aggregate stability even at deep soil layer.

## Conclusion

The effects of HM contamination on AMF status, soil properties, aggregate distribution and stability, and their correlations at different soil depths were comprehensively revealed in the current study. Total/DTPA-extractable Pb had significant inhibitory effects on MC and HLD at 0–20 cm soil depth, however, AMF propagules never disappeared completely even in soils contaminated by high concentrations of Pb. The results showed that excessive PbPb had harmful impacts on AMF growth, but at the same time, certain species of AMF likely had developed mechanisms that allow them to survive in HM contaminated soils. T-GRSP, EE-GRSP, SOM and SOC concentrations were significantly inhibited by Pb compared to Zn at 0–20 cm soil depth, indicating that HM presented hazardous effects on soil properties and that Pb exhibited greater toxicity than Zn at shallow layer of soil (0–20 cm). In addition, GRSP, SOM and SOC were beneficial to WSA_<2000 μm_ and MWD, and had significantly positive correlations with MC, HLD at 0–20 cm soil depth. The results indicated that AMF played an essential role in aggregate distribution and stability in HM polluted soils. The use of AMF as an additional biotechnological tool to enhance the recovery of HM polluted soils probably provides an effective way for phytoremediation. However, further studies are required to evaluate the exact role of plant-AMF combination in aggregate distribution and stability on larger scales and under more complex environmental conditions.

## Supporting information

S1 Fig Simplified location map showing the five sampling sites of the study area. (PDF)

S2 Fig The typical structures of arbuscular mycorrhizal fungi (AMF) in the roots of *S*. *viciifolia* at S1 (a) and S5 (b). (PDF)

S3 Fig The availability of Pb and Zn in heavy metal contaminated soil. (PDF)

S4 Fig Correlations among pH (a), SOM (b) and HM availability of soil samples at different soil depths and study sites. (PDF)

S5 Fig Leaf net photosynthetic rate (Pn) and chlorophyll (Chl) concentration of *S*. *viciifolia* grown at different study sites. (PDF)

S1 Table Soil chemical properties at different soil depths and study sites. (PDF)

S2 Table Correlational analysis among soil properties, HM concentrations and availabilities in rhizosphere soil at different soil depths and study sites. (PDF)

S3 Table Correlational analysis among AMF status, GRSP concentration and soil properties at different soil depths and study sites. (PDF)

S4 Table Correlational analysis among AMF status, GRSP concentration, HM concentration and HM availability at different soil depths and study sites. (PDF)

S5 Table Correlations among soil aggregate distribution, AMF status, GRSP concentration and soil properties at different soil depths. (PDF)
