# Supplementary figures and images for: The effects of arbuscular mycorrhizal fungi on glomalin-related soil protein distribution, aggregate stability and their relationships with soil properties at different soil depths in lead-zinc contaminated area
Source: PLoS One. 2017 Aug 3;12(8):e0182264. doi: 10.1371/journal.pone.0182264 (PMC5542611; doi:10.1371/journal.pone.0182264)

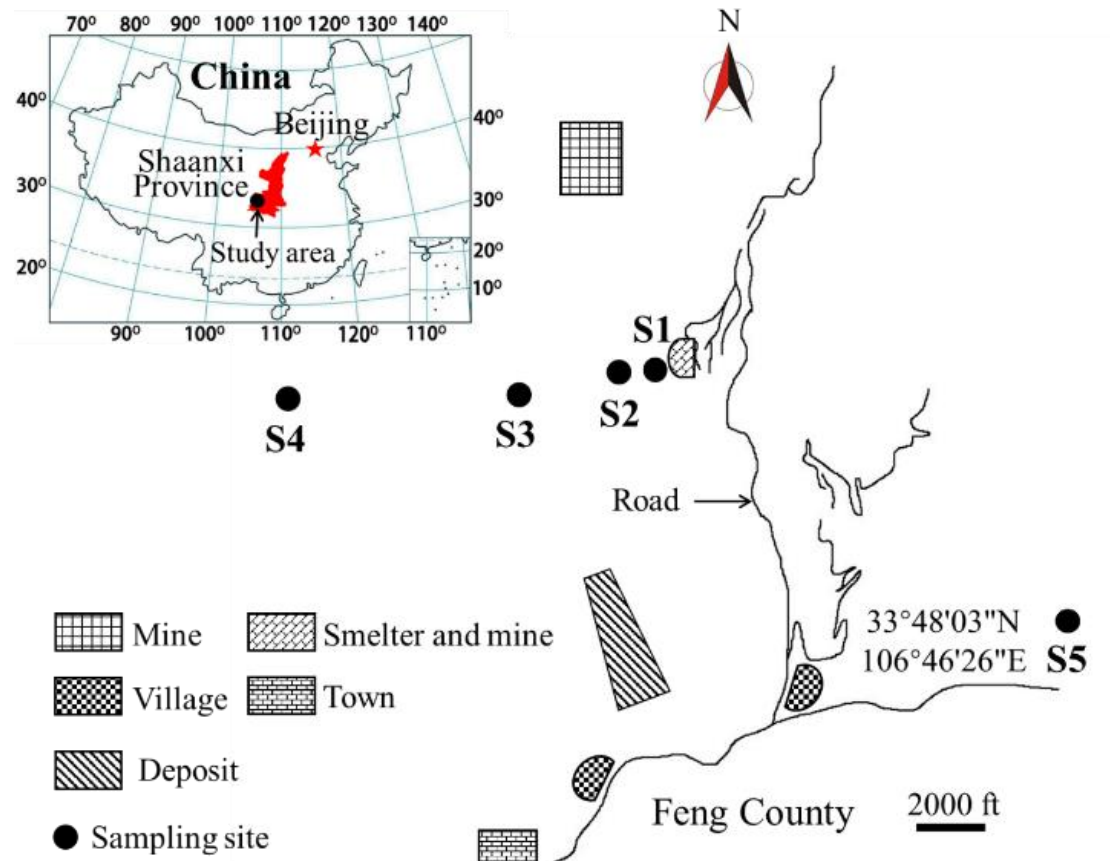

**S1 Fig.** Simplified location map showing the five sampling sites of the study area.

Supplement: S1 Fig — (PDF) [file pone.0182264.s001.pdf]
