# Supplementary material for: The effects of arbuscular mycorrhizal fungi on glomalin-related soil protein distribution, aggregate stability and their relationships with soil properties at different soil depths in lead-zinc contaminated area
Source: PLoS One. 2017 Aug 3;12(8):e0182264. doi: 10.1371/journal.pone.0182264 (PMC5542611; doi:10.1371/journal.pone.0182264)
Supplement: S2 Fig — (PDF) [file pone.0182264.s002.pdf]

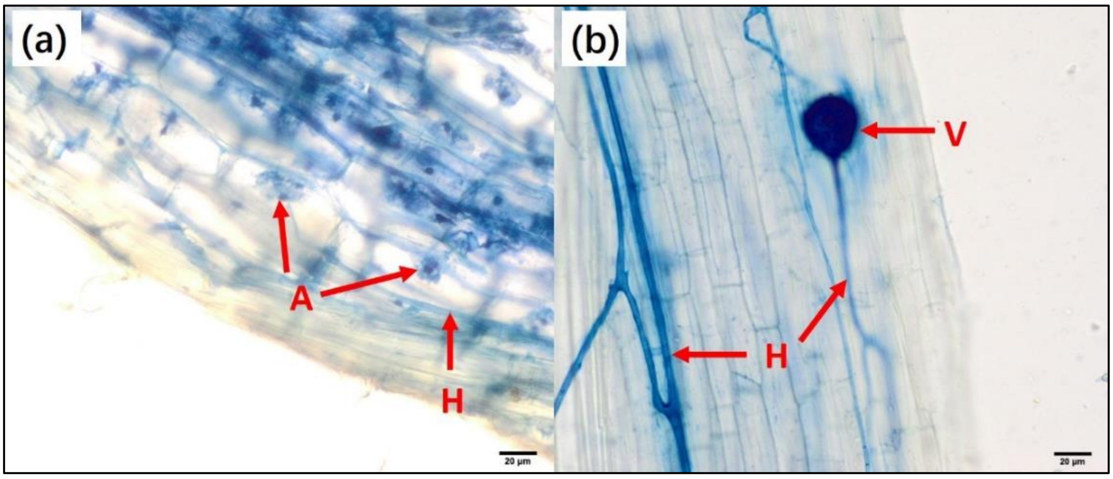

S2 Fig. The typical structures of arbuscular mycorrhizal fungi (AMF) in the roots of *Sophora viciifolia* at S1 (a) and S5 (b). A, arbuscules; V, vesicles; H, hyphae. Bars present the length of 20 µm for the images.
