# Supplementary material for: The effects of arbuscular mycorrhizal fungi on glomalin-related soil protein distribution, aggregate stability and their relationships with soil properties at different soil depths in lead-zinc contaminated area
Source: PLoS One. 2017 Aug 3;12(8):e0182264. doi: 10.1371/journal.pone.0182264 (PMC5542611; doi:10.1371/journal.pone.0182264)
Supplement: S3 Fig — (PDF) [file pone.0182264.s003.pdf]

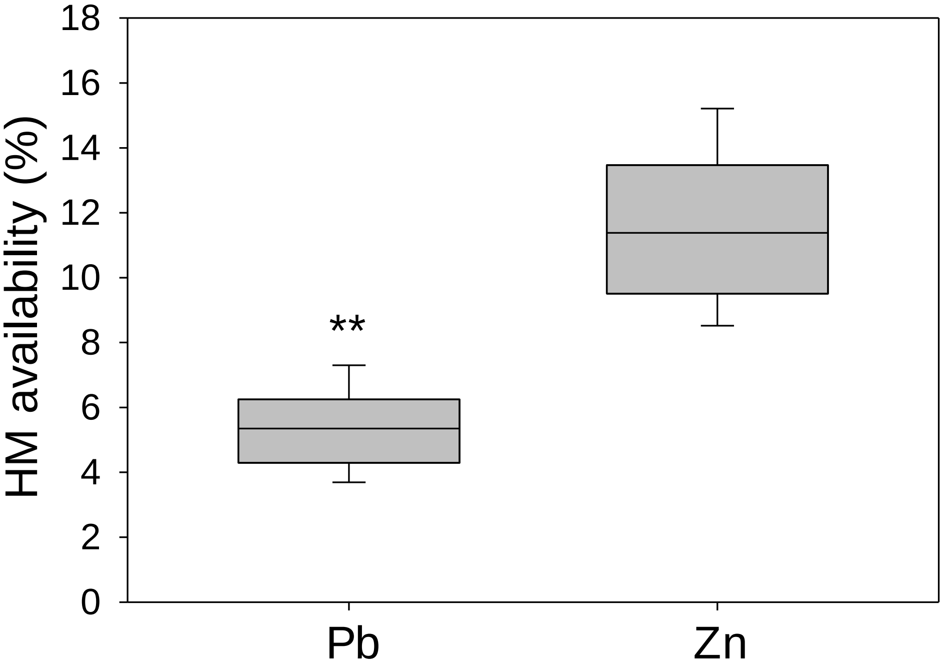

S3 Fig. The availability of Pb and Zn in heavy metal contaminated soil. ** Indicates significant differences between Pb and Zn availability ($P < 0.01$ from paired t-tests, n = 6).
