# Supplementary material for: The effects of arbuscular mycorrhizal fungi on glomalin-related soil protein distribution, aggregate stability and their relationships with soil properties at different soil depths in lead-zinc contaminated area
Source: PLoS One. 2017 Aug 3;12(8):e0182264. doi: 10.1371/journal.pone.0182264 (PMC5542611; doi:10.1371/journal.pone.0182264)
Supplement: S4 Fig — (PDF) [file pone.0182264.s004.pdf]

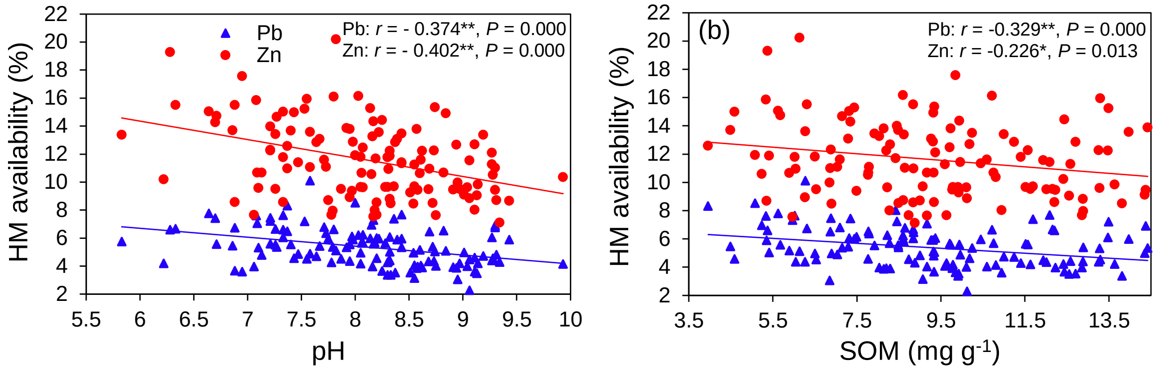

S4 Fig. Correlations among pH (a), SOM (b) and HM availability of soil samples at different soil depths and study sites. Linear regression fits and associated *r* and *P* values are given in each panel.
