# Supplementary material for: The effects of arbuscular mycorrhizal fungi on glomalin-related soil protein distribution, aggregate stability and their relationships with soil properties at different soil depths in lead-zinc contaminated area
Source: PLoS One. 2017 Aug 3;12(8):e0182264. doi: 10.1371/journal.pone.0182264 (PMC5542611; doi:10.1371/journal.pone.0182264)
Supplement: S5 Fig — (PDF) [file pone.0182264.s005.pdf]

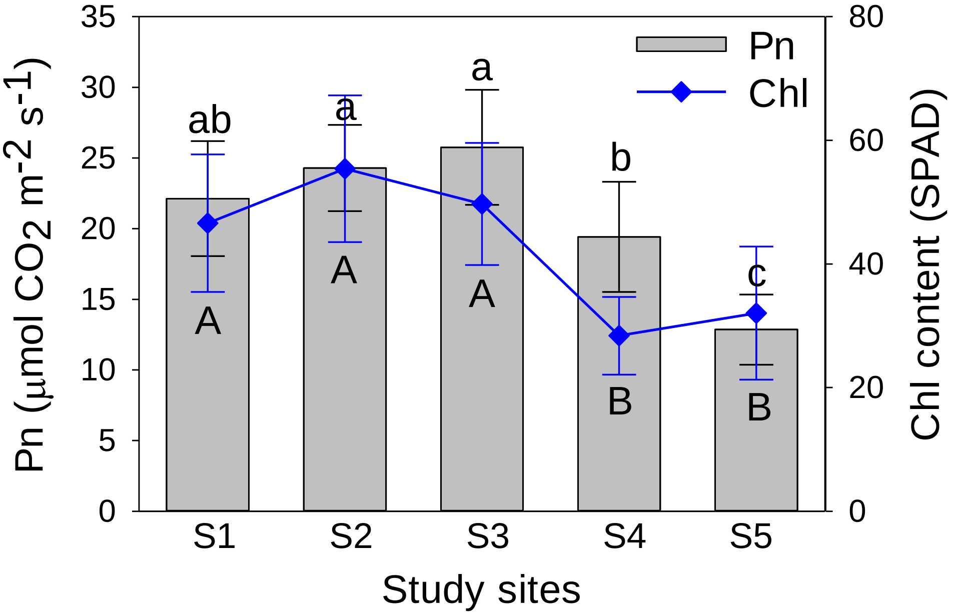

S5 Fig. Leaf net photosynthetic rate (Pn) and chlorophyll (Chl) concentration of *S. viciifolia* grown at different study sites. Each value is the mean ± SD (n = 6). Different letters indicate statistically significant differences (one-way ANOVA followed by SNK test, $P < 0.05$) at five study sites (S1, S2, S3, S4 and S5).
