# Supplementary material for: The effects of arbuscular mycorrhizal fungi on glomalin-related soil protein distribution, aggregate stability and their relationships with soil properties at different soil depths in lead-zinc contaminated area
Source: PLoS One. 2017 Aug 3;12(8):e0182264. doi: 10.1371/journal.pone.0182264 (PMC5542611; doi:10.1371/journal.pone.0182264)
Supplement: S1 Table — (PDF) [file pone.0182264.s006.pdf]

**S1 Table.** Soil chemical properties at different soil depths and study sites.

| Sites | Depth | Pb concentration | | Zn concentration | |
|---|---|---|---|---|---|
| | (cm) | TPb | DPb | TZn | DZn |
| S1 | 0-10 | 69.6±13.4a | 3.42±0.68a | 156±43.7a | 15.1±3.95a |
| | 10-20 | 55.8±8.29b | 3.05±0.20a | 127±35.1a | 13.6±1.66a |
| | 20-30 | 35.4±8.10bc | 1.66±0.18b | 116±25.1a | 11.6±1.31a |
| | 30-40 | 47.0±10.7c | 2.03±0.27b | 134±20.1a | 12.7±1.62a |
| S2 | 0-10 | 263±62.4ab | 12.6±1.21b | 340±70.5ab | 39.9±4.95b |
| | 10-20 | 297±62.0a | 14.7±1.97a | 392±65.7a | 50.5±7.55a |
| | 20-30 | 234±56.4bc | 8.95±1.61c | 275±44.2b | 30.3±7.44c |
| | 30-40 | 150±33.6c | 6.63±0.72d | 293±34.3b | 26.3±4.29c |
| S3 | 0-10 | 450±80.0a | 24.0±2.08a | 658±111a | 72.9±5.13ab |
| | 10-20 | 466±126a | 22.1±2.77a | 608±101a | 76.4±13.4a |
| | 20-30 | 216±30.6b | 8.85±1.49b | 668±101a | 72.6±9.37ab |
| | 30-40 | 234±51.1b | 8.78±1.22b | 626±35.4a | 60.4±7.49b |
| S4 | 0-10 | 2338±340a | 129±16.1a | 265±44.9a | 31.5±2.49ab |
| | 10-20 | 2395±643a | 144±16.7a | 309±42.8a | 33.5±6.09a |
| | 20-30 | 1893±323a | 107±27.5b | 250±55.8a | 28.2±4.23ab |
| | 30-40 | 1339±455b | 66.2±9.65c | 249±58.1a | 24.6±6.06b |
| S5 | 0-10 | 5319±449a | 380±51.6a | 311±79.5a | 41.7±4.47ab |
| | 10-20 | 5636±653a | 362±41.5a | 338±67.2a | 49.0±8.99a |
| | 20-30 | 3934±790b | 267±27.8b | 299±61.7a | 40.1±5.80b |
| | 30-40 | 2915±233c | 178±16.3c | 195±68.3b | 24.9±3.27c |

SOM, soil organic matter; SOC, soil organic carbon; TN, total nitrogen; TP, total phosphorus. Each value is the mean ± SD (n = 6). Different letters indicate statistically significant differences (one-way ANOVA followed by SNK test, $P < 0.05$) at four soil depths (0-10, 10-20, 20-30, 30-40 cm).
