# Supplementary material for: The effects of arbuscular mycorrhizal fungi on glomalin-related soil protein distribution, aggregate stability and their relationships with soil properties at different soil depths in lead-zinc contaminated area
Source: PLoS One. 2017 Aug 3;12(8):e0182264. doi: 10.1371/journal.pone.0182264 (PMC5542611; doi:10.1371/journal.pone.0182264)
Supplement: S2 Table — (PDF) [file pone.0182264.s007.pdf]

S2 Table. Correlational analysis among soil properties, HM concentrations and availabilities in rhizosphere soil at different soil depths and study sites.

	Depth (cm)	HM concentrations				HM availabilities	
		TPb	DPb	TZn	DZn	Pb	Zn
HM concentrations							
T-Pb	0-10	1	**0.981****	-0.128NS	0.006NS	**0.562****	**0.402***
	10-20	1	**0.983****	-0.042NS	0.044NS	0.281NS	0.243NS
	20-30	1	**0.951****	-0.121NS	0.017NS	**0.578****	**0.507***
	30-40	1	**0.979****	-0.334NS	-0.182NS	**0.483****	**0.638****
	0-40	1	**0.975****	-0.104NS	0.045NS	**0.478****	**0.429****
D-Pb	0-10	**0.981****	1	-0.117NS	0.012NS	**0.646****	**0.381***
	10-20	**0.983****	1	-0.061NS	0.041NS	**0.391***	0.281NS
	20-30	**0.951****	1	-0.100NS	0.035NS	**0.764****	**0.470****
	30-40	**0.979****	1	-0.322NS	-0.172NS	**0.590****	**0.600****
	0-40	**0.975****	1	-0.095NS	0.054NS	**0.591****	**0.418****
T-Zn	0-10	-0.128NS	-0.117NS	1	**0.901****	0.059NS	-0.227NS
	10-20	-0.042NS	-0.061NS	1	**0.913****	-0.185NS	0.016NS
	20-30	-0.121NS	-0.100NS	1	**0.946****	-0.162NS	-0.120NS
	30-40	-0.334NS	-0.322NS	1	**0.940****	-0.288NS	-0.306NS
	0-40	-0.104NS	-0.095NS	1	**0.911****	-0.106NS	-0.122NS
D-Zn	0-10	0.006NS	0.012NS	**0.901****	1	0.119NS	0.168NS
	10-20	0.044NS	0.041NS	**0.913****	1	-0.081NS	**0.380***
	20-30	0.017NS	0.035NS	**0.946****	1	-0.074NS	0.165NS
	30-40	-0.182NS	-0.172NS	**0.940****	1	-0.169NS	-0.030NS
	0-40	0.045NS	0.054NS	**0.911****	1	0.018NS	0.243**
Soil properties							
pH	0-10	**-0.546****	**-0.541****	0.187NS	0.111NS	**-0.374***	**-0.372***
	10-20	**-0.580****	**-0.595****	0.121NS	-0.102NS	**-0.396***	**-0.608****
	20-30	**-0.569****	**-0.566****	0.043NS	-0.048NS	**-0.449***	**-0.472****
	30-40	-0.238NS	-0.248NS	-0.002NS	0.013NS	-0.203NS	-0.118NS
	0-40	**-0.514****	**-0.515****	0.071NS	-0.047NS	**-0.374****	**-0.402****
SOM	0-10	**-0.728****	**-0.731****	0.298NS	0.086NS	**-0.464****	**-0.506****
	10-20	**-0.659****	**-0.704****	**-0.450***	0.300NS	0.225NS	-0.248NS
	20-30	**-0.686****	**-0.651****	**0.465****	0.350NS	**-0.480****	-0.311NS
	30-40	-0.222NS	-0.212NS	0.297NS	0.212NS	-0.284NS	-0.237NS
	0-40	**-0.538****	**-0.547****	**0.350****	**0.266****	**-0.329****	**-0.226***
SOC	0-10	**-0.772****	**-0.767****	0.220NS	0.067NS	**-0.447***	**-0.402***
	10-20	**-0.544****	**-0.594****	**0.367***	0.173NS	**-0.613****	**-0.440***
	20-30	-0.325NS	-0.361NS	0.203NS	0.111NS	-0.325NS	-0.269NS
	30-40	**-0.490****	**-0.547****	**0.459***	**0.426***	**-0.512****	-0.344NS
	0-40	**-0.426****	**-0.450****	**0.323****	**0.253****	**-0.315****	**-0.220****

TPb, total Pb; TZn, total Zn; DPb, DTPA-extractable Pb; DZn, DTPA-extractable Zn; SOM, soil organic matter; SOC, soil organic carbon. $^{**}P < 0.01$; $^{*}P < 0.05$; NS, not significant.
