# Supplementary material for: The effects of arbuscular mycorrhizal fungi on glomalin-related soil protein distribution, aggregate stability and their relationships with soil properties at different soil depths in lead-zinc contaminated area
Source: PLoS One. 2017 Aug 3;12(8):e0182264. doi: 10.1371/journal.pone.0182264 (PMC5542611; doi:10.1371/journal.pone.0182264)
Supplement: S3 Table — (PDF) [file pone.0182264.s008.pdf]

**S3 Table.** Correlational analysis among AMF status, GRSP concentration and soil properties at different soil depths and study sites.

| | Depth (cm) | Soil properties pH | SOM | SOC | TN | TP |
|---|---|---|---|---|---|---|
| AMF status | | | | | | |
| MC | 0-10 | **0.423*** | **0.376*** | **0.507**** | 0.306NS | **0.416*** |
| | 10-20 | 0.345NS | **0.515**** | **0.488**** | 0.303NS | 0.229NS |
| | 20-30 | 0.263NS | 0.206NS | 0.034NS | 0.007NS | 0.324NS |
| | 30-40 | 0.327NS | 0.066NS | 0.215NS | -0.094NS | 0.289NS |
| | 0-40 | 0.145NS | **0.421**** | **0.521**** | **0.464**** | **0.578**** |
| SP | 0-10 | 0.316NS | -0.080NS | -0.060NS | -0.055NS | 0.017NS |
| | 10-20 | -0.037NS | -0.246NS | -0.173NS | -0.079NS | 0.137NS |
| | 20-30 | 0.085NS | **0.489**** | 0.294NS | 0.197NS | 0.357NS |
| | 30-40 | -0.094NS | -0.261NS | 0.334NS | 0.281NS | 0.256NS |
| | 0-40 | 0.002NS | 0.109NS | **0.238*** | **0.250**** | **0.400**** |
| HLD | 0-10 | 0.276NS | **0.367*** | **0.534**** | **0.414*** | **0.407*** |
| | 10-20 | **0.461*** | **0.502**** | 0.225NS | 0.356NS | **0.482**** |
| | 20-30 | 0.335NS | 0.123NS | 0.262NS | 0.193NS | 0.176NS |
| | 30-40 | -0.287NS | 0.055NS | -0.097NS | 0.077NS | 0.061NS |
| | 0-40 | 0.122NS | **0.388**** | **0.405**** | **0.470**** | **0.523**** |
| GRSP concentration | | | | | | |
| T-GRSP | 0-10 | 0.299NS | **0.425*** | **0.532**** | **0.472**** | 0.359NS |
| | 10-20 | **0.424*** | **0.500**** | **0.530**** | **0.472**** | 0.249NS |
| | 20-30 | 0.341NS | **0.558**** | **0.476**** | **0.397*** | **0.409*** |
| | 30-40 | **0.515**** | 0.079NS | **0.606**** | 0.198NS | 0.343NS |
| | 0-40 | **0.269*** | **0.486**** | **0.618**** | **0.543**** | **0.510**** |
| EE-GRSP | 0-10 | 0.276NS | 0.300NS | **0.433*** | 0.247NS | 0.322NS |
| | 10-20 | **0.415*** | **0.519**** | **0.568**** | **0.439*** | 0.333NS |
| | 20-30 | -0.019NS | **0.474**** | **0.460*** | 0.184NS | **0.365*** |
| | 30-40 | 0.105NS | -0.072NS | **0.389*** | 0.027NS | **0.569**** |
| | 0-40 | 0.103NS | **0.446**** | **0.587**** | **0.473**** | **0.572**** |

MC, mycorrhizal colonization; SP, spore density; HLD, hyphal length density; T-GRSP, total GRSP concentration; EE-GRSP, easily extractable GRSP concentration; SOM, soil organic matter; SOC, soil organic carbon; TN, total nitrogen; TP, total phosphorus. $^{**}P < 0.01$; $^{*}P < 0.05$; NS, not significant.
