# Supplementary material for: The effects of arbuscular mycorrhizal fungi on glomalin-related soil protein distribution, aggregate stability and their relationships with soil properties at different soil depths in lead-zinc contaminated area
Source: PLoS One. 2017 Aug 3;12(8):e0182264. doi: 10.1371/journal.pone.0182264 (PMC5542611; doi:10.1371/journal.pone.0182264)
Supplement: S4 Table — (PDF) [file pone.0182264.s009.pdf]

S4 Table. Correlational analysis among AMF status, GRSP concentration, HM concentration and HM availability at different soil depths and study sites.

	Depth	HM concentration				HM availability	
	(cm)	TPb	DPb	TZn	DZn	Pb	Zn
AMF status							
MC	0-10	**-0.616****	**-0.619****	0.047NS	0.069NS	-0.234NS	0.047NS
	10-20	**-0.603****	**-0.614****	0.169NS	0.031NS	-0.271NS	-0.282NS
	20-30	-0.305NS	-0.226NS	0.061NS	0.070NS	-0.094NS	-0.056NS
	30-40	-0.065NS	-0.039NS	**0.394***	**0.412***	-0.054NS	-0.100NS
	0-40	**-0.194***	**-0.183***	**0.193***	**0.260***	0.055NS	0.131NS
SP	0-10	-0.236NS	-0.254NS	**-0.423***	-0.359NS	-0.306NS	0.020NS
	10-20	0.268NS	0.273NS	-0.068NS	-0.015NS	0.063NS	0.146NS
	20-30	**-0.499****	**-0.489****	0.154NS	0.149NS	**-0.395***	-0.113NS
	30-40	-0.087NS	-0.082NS	0.256NS	0.296NS	0.027NS	0.038NS
	0-40	0.006NS	0.008NS	0.050NS	0.138NS	-0.013NS	0.162NS
HLD	0-10	**-0.393***	**-0.396***	0.293NS	0.321NS	-0.187NS	0.024NS
	10-20	**-0.371***	**-0.379***	0.092NS	-0.122NS	-0.129NS	**-0.515****
	20-30	**-0.390***	**-0.427***	-0.013NS	-0.123NS	**-0.471****	-0.338NS
	30-40	-0.122NS	-0.105NS	0.343NS	0.274NS	-0.136NS	-0.214NS
	0-40	-0.178NS	**-0.182***	**0.205***	**0.188***	-0.053NS	-0.049NS
GRSP concentration							
T-GRSP	0-10	**-0.423***	**-0.455***	**0.505****	**0.511****	**-0.416***	-0.001NS
	10-20	**-0.596****	**-0.609****	0.244NS	0.155NS	**-0.388***	-0.109NS
	20-30	**-0.533****	**-0.516****	0.315NS	0.173NS	**-0.377***	**-0.434***
	30-40	-0.218NS	-0.300NS	0.195NS	0.180NS	**-0.383***	-0.174NS
	0-40	**-0.307****	**-0.315***	**0.325****	**0.332****	**-0.199***	0.009NS
EE-GRSP	0-10	**-0.380***	**-0.362***	0.316NS	0.293NS	0.123NS	-0.041NS
	10-20	**-0.424***	**-0.491****	**0.406***	0.278NS	**-0.601****	-0.221NS
	20-30	-0.318NS	-0.323NS	**0.380***	0.260NS	-0.260NS	-0.312NS
	30-40	-0.307NS	-0.356NS	0.321NS	0.189NS	**-0.395***	-0.156NS
	0-40	**-0.188***	**-0.201***	**0.349****	**0.341****	-0.096NS	0.018NS

TPb, total Pb; TZn, total Zn; DPb, DTPA-extractable Pb; DZn, DTPA-extractable Zn. ***P* < 0.01; **P* < 0.05; NS, not significant.
