# Supplementary material for: The effects of arbuscular mycorrhizal fungi on glomalin-related soil protein distribution, aggregate stability and their relationships with soil properties at different soil depths in lead-zinc contaminated area
Source: PLoS One. 2017 Aug 3;12(8):e0182264. doi: 10.1371/journal.pone.0182264 (PMC5542611; doi:10.1371/journal.pone.0182264)
Supplement: S5 Table — (PDF) [file pone.0182264.s010.pdf]

S5 Table. Correlations among soil aggregate distribution, AMF status, GRSP concentration and soil properties at different soil depths.

Depth	Soil	AMF status			GRSP		Soil properties								
(cm)	aggregates	MC	SP	HLD	T-GRSP	EE-GRSP	pH	SOM	SOC	TN	TP	TPb	DPb	TZn	DZn
0-10	$WSA_{>2000\ \mu m}$	NS	NS	NS	NS	+*	+*	+*	+**	NS	NS	-*	-**	NS	NS
	$WSA_{2000\text{-}250\ \mu m}$	NS	NS	NS	NS	NS	NS	NS	NS	NS	NS	NS	NS	NS	NS
	$WSA_{250\text{-}53\ \mu m}$	NS	NS	NS	NS	NS	NS	NS	NS	NS	NS	NS	NS	NS	NS
	$WSA_{<53\ \mu m}$	-**	NS	-**	-**	-**	-**	-**	-**	-**	-**	-**	-**	NS	NS
	MWD	+*	NS	NS	+*	+*	+*	+**	+**	+*	NS	-**	-**	+*	NS
10-20	$WSA_{>2000\ \mu m}$	NS	NS	NS	+**	+*	NS	NS	NS	NS	NS	-*	-**	NS	NS
	$WSA_{2000\text{-}250\ \mu m}$	NS	-*	NS	NS	NS	NS	NS	NS	NS	NS	-**	-**	NS	NS
	$WSA_{250\text{-}53\ \mu m}$	NS	NS	NS	NS	NS	NS	NS	NS	NS	NS	NS	NS	NS	NS
	$WSA_{<53\ \mu m}$	NS	NS	NS	NS	NS	-*	-**	-*	-**	NS	+**	+**	NS	NS
	MWD	+*	NS	NS	+**	+*	NS	+**	+*	+*	NS	-**	-**	NS	NS
20-30	$WSA_{>2000\ \mu m}$	NS	+**	NS	NS	NS	NS	NS	NS	NS	NS	-*	-*	NS	NS
	$WSA_{2000\text{-}250\ \mu m}$	NS	+*	NS	NS	NS	NS	NS	NS	NS	NS	NS	NS	NS	NS
	$WSA_{250\text{-}53\ \mu m}$	NS	-*	-*	NS	NS	NS	NS	NS	NS	NS	+*	NS	NS	NS
	$WSA_{<53\ \mu m}$	NS	NS	NS	NS	-*	NS	NS	-*	NS	NS	NS	NS	NS	NS
	MWD	NS	+**	NS	+*	+*	NS	+**	NS	NS	NS	-*	-**	NS	NS
30-40	$WSA_{>2000\ \mu m}$	NS	NS	NS	NS	+*	NS	NS	+*	NS	NS	NS	NS	NS	NS
	$WSA_{2000\text{-}250\ \mu m}$	NS	NS	NS	NS	NS	NS	NS	NS	NS	NS	NS	NS	NS	NS
	$WSA_{250\text{-}53\ \mu m}$	NS	NS	NS	NS	NS	NS	-*	NS	NS	NS	+*	+*	NS	NS
	$WSA_{<53\ \mu m}$	NS	NS	NS	NS	NS	NS	NS	NS	NS	NS	NS	NS	NS	NS
	MWD	NS	NS	NS	+*	NS	NS	NS	+*	NS	NS	NS	NS	NS	NS

MC, mycorrhizal colonization; SP, spore density; HLD, hyphal length density; T-GRSP, total GRSP concentration; EE-GRSP, easily extractable GRSP concentration; SOM, soil organic matter; SOC, soil organic carbon; TN, total nitrogen; TPb, total Pb; TZn, total Zn; DPb, DTPA-extractable Pb; DZn, DTPA-extractable Zn. +, positive correlation; -, negative correlation; $^{**}P < 0.01$; $^{*}P < 0.05$; NS, not significant.
